# Artificial light at night reduces earthworm activity but increases growth of invasive ragweed

**DOI:** 10.1186/s12862-024-02200-x

**Published:** 2024-01-19

**Authors:** Marion Mittmannsgruber, Zenia Kavassilas, Bernhard Spangl, Edith Gruber, Elias Jagg, Johann G. Zaller

**Affiliations:** 1 Department of Integrative Biology and Biodiversity Research, Institute of Zoology, University of Natural Resources and Life Sciences Vienna (BOKU), Vienna, 1180 Austria; 2 Department of Landscape, Spatial and Infrastructure Sciences, Institute of Statistics, University of Natural Resources and Life Sciences Vienna (BOKU), Vienna, 1180 Austria

**Keywords:** Light pollution, Earthworms, *Lumbricus terrestris*, Ragweed, *Ambrosia artemisiifolia*, Agroecology, Plant-animal interactions, Artificial light at night, ALAN

## Abstract

**Background:**

Artificial light at night, also referred to as light pollution (LP), has been shown to affect many organisms. However, little is known about the extent to which ecological interactions between earthworms and plants are altered by LP. We investigated the effects of LP on anecic earthworms (*Lumbricus terrestris*) that come to the surface at night to forage and mate, and on the germination and growth of the invasive and allergenic ragweed (*Ambrosia artemisiifolia).* In a full factorial pot experiment in the greenhouse, we tested four factors and their interactions: LP (5 lux vs. 0 lux at night), earthworms (two individuals vs. none), plant species (seeding of ragweed only vs. mixed with *Phacelia* seeds) and sowing depth (seed placed at the surface vs. in 5 cm depth). Data were analysed using Generalized Linear (Mixed) Models and multifactorial ANOVAs with soil parameters as covariates.

**Results:**

Light pollution reduced earthworm surface activity by 76% as measured by casting activity and toothpick index; 85% of mating earthworms were observed in the absence of LP. Light pollution in interaction with earthworms reduced ragweed germination by 33%. However, LP increased ragweed height growth by 104%. Earthworms reduced ragweed germination especially when seeds were placed on the soil surface, suggesting seed consumption by earthworms.

**Conclusions:**

Our data suggest that anecic earthworms are negatively affected by LP because reduced surface activity limits their ability to forage and mate. The extent to which earthworm-induced ecosystem services or community interactions are also affected by LP remains to be investigated. If the increased height growth of ragweed leads to increased pollen and seed production, it is likely that the competition of ragweed with field crops and the risks to human health will also increase under LP.

**Supplementary Information:**

The online version contains supplementary material available at 10.1186/s12862-024-02200-x.

## Background

Artificial light at night, or light pollution is the brightening of the night sky by anthropogenic light sources [1]. In general, urban areas are affected by light pollution to a greater extent than rural areas [2]. Light pollution has increased worldwide in recent decades, by 49% since 1992 [3]. The reasons for this increase are primarily due to increasing urbanization, followed by agriculture and industry [4], whereby the illuminated areas are constantly expanding and the brightness of the already illuminated areas continues to increase [5]. There is also a difference between a brief, direct light (e.g., from vehicle headlights) and the chronic brightening of the night by skyglow [6]. Skyglow is the diffuse, low-intensity illumination often found in and around urban centres when light escapes into the atmosphere [7]. It causes a chronic increase in nighttime brightness in the area and can spread several kilometres from the light source [2]. It is estimated that 23% of the world’s land area is affected by light pollution, with Europe and North America being the most affected, accounting for 90% and 50% of the land area, respectively [8].

Light pollution has been found to affect many different organisms, including humans [9], a variety of other animal species [6, 10], and plants [11]. For plants, light is a very important resource as it not only forms the basis for photosynthesis, but also provides information that affects plant growth and phenology [11] [12]. For example, it has been found that budburst of various tree species occurs earlier under light pollution [12], while herbaceous plants (e.g., *Lotus pedunculatus*) produce fewer inflorescences under light pollution [13].

In animals, the effects of light pollution have been studied extensively for several taxa such as birds [14–16], mammals [17, 18], and especially insects [19–22]. However, the effects on soil-dwelling organisms have only rarely been studied [23, 24]. Although the effects of light pollution range from lethal [21] to merely behavioural [14], they all have the potential to alter the composition of soil communities [7, 15, 19, 22, 23, 25]. In addition, interactions between different organisms, such as plants and pollinating insects, can also be affected. Moths that pollinate at night have been found to carry a lower pollen load in illuminated areas than in unlit areas [26], and pollination by nocturnal insects in general has been found to decrease by more than 60% with light pollution, which also reduces fruit production [27].

One group of animals whose response to light pollution has rarely been studied so far are earthworms. Although most earthworms live permanently in the soil and are therefore not directly affected by light pollution, anecic earthworms such as *Lumbricus terrestris* forage at night on the soil surface [28], where they also mate [29] and where they are potentially vulnerable to light pollution. To our knowledge, only one study has shown that light pollution affects the nocturnal surface activity of anecic earthworms [24], but even Charles Darwin already noted in the 19th century that earthworms react negatively to light and retreat into their burrows [28]. Earthworms perceive light through photoreceptor cells in their epidermis, which are particularly abundant in the anterior part of the body [30]. The effects of light pollution on earthworms could affect their importance as ecosystem engineers [31], soil structure through their burrowing activity [32] and plant growth promoting effects [33]. Earthworms can also directly affect seed dispersal and germination by consuming seeds [34] and removing seeds from the soil surface [35] or transporting them within the soil [36]. Seeds can even be transported to deeper soil layers where successful germination is less likely, while seeds from deeper soil layers can be transported to the soil surface and only germinate due to earthworm activity [36]. It is also known that earthworms prefer the seeds of different plants when given a choice [34], and that they may alter plant communities through this behaviour [37].

In one study, *L. terrestris* was found to specifically interfere with the establishment of giant ragweed (*Ambrosia trifida*), by burying ragweed seeds, resulting in a nearly 40% reduction in seedling emergence [35]. While this reduces weed emergence in the short term, the seeds enter the soil seedbank where they can survive in the long term [35]. Earthworms also compete with other seed predators and may protect ragweed seeds from being consumed by predators [38]. In Europe, a related ragweed species with rather similar seed characteristics [39], common ragweed (*Ambrosia artemisiifolia*), became an invasive neophyte several decades ago [40]. Apart from the economic impact of common ragweed through reduced crop yields [41], its pollen can also be highly allergenic [42].

Invasive plant species have been found to increase their relative biomass production in plant communities under light pollution [43], however, it is not known whether this is also the case for common ragweed. Additionally, more common plant species benefit from light pollution compared to rare species [44], potentially contributing to the loss of endangered plant species.

This study investigated the effects of light pollution on the interaction between the activity of anecic earthworms (*L. terrestris)* and germination and growth of common ragweed (*A. artemisiifolia)*. We focused on the following hypotheses: (i) Light pollution leads to a reduction in the nocturnal surface activity of *L. terrestris* [24], (ii) Reduced earthworm surface activity due to light pollution benefits ragweed by reducing the detrimental effects of earthworms on ragweed [35, 47], and (iii) Light pollution increases ragweed growth and biomass production by providing additional light resources [11, 43]. These hypotheses were tested in in a full-factorial greenhouse experiment. We set up experimental units containing only ragweed and mixed units with a cover crop (*Phacelia tanacetifolia)* in which ragweed could potentially emerge.

## Results

### Earthworm activity

**Fig. 1:**
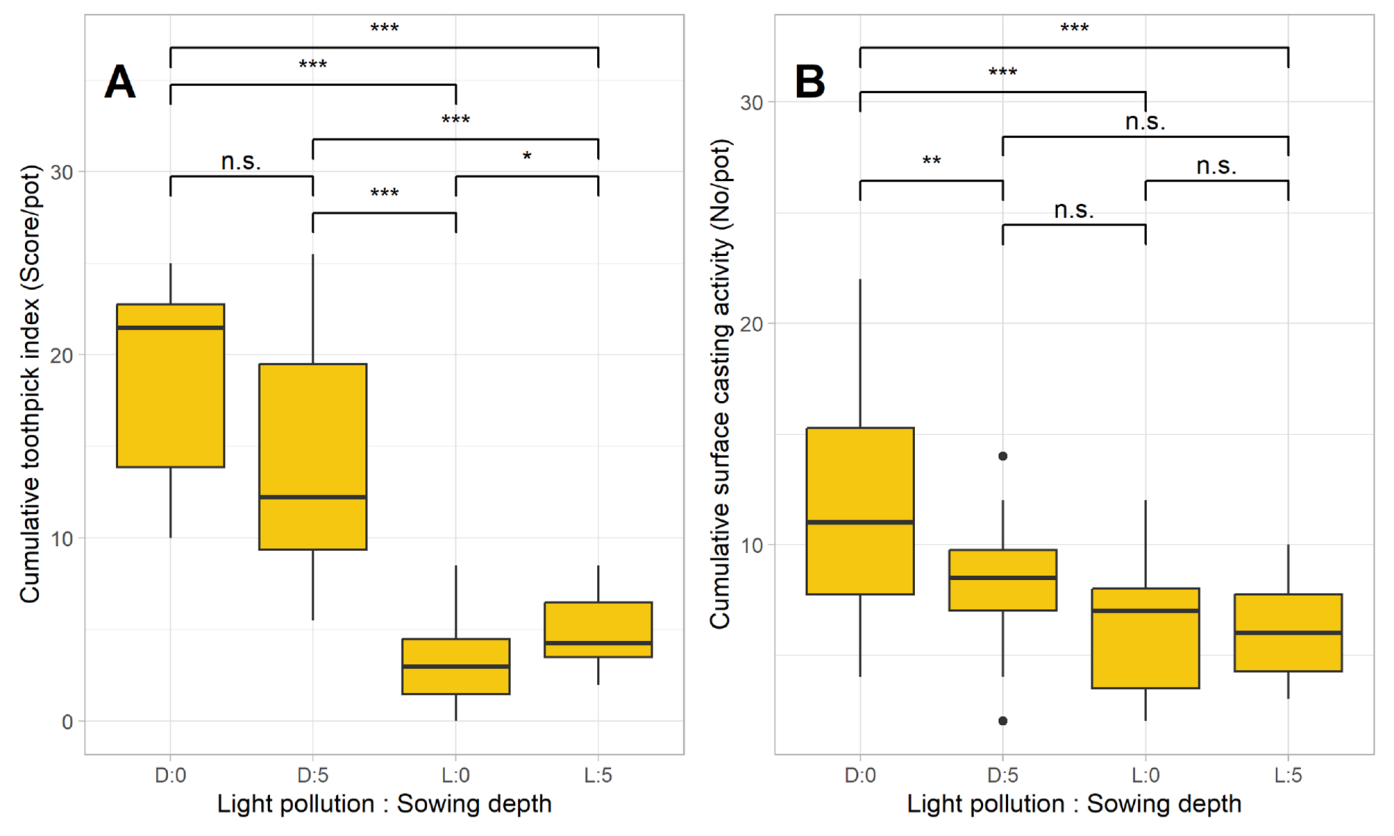
Earthworm surface activity assessed with the toothpick index (**A**) and the surface casting activity (**B**) throughout all 12 samplings, considering effects of light pollution (D…dark, L…light) and sowing depth (0…surface sown, 5…sown in 5 cm depth). *N* = 6. Each box represents the 1st and 3rd quartiles, the median as the horizontal line and the whiskers as minimum and maximum values

Light pollution resulted in a 76% decrease in nocturnal surface activity as measured by the toothpick index and a 37% decrease in surface casting activity (Fig. 1; Table 1). Light pollution and sowing depth interactively affected both the toothpick index and surface casting (Table 1): In the absence of light pollution, earthworm casting activity was highest when seeds were surface sown, whereas under light pollution, earthworm surface activity (toothpicks) tended to be higher when seeds were buried (Fig. 1).

It was also found that several of the soil parameters included as covariates in the analyses had significant effects on earthworm activity (Table 1). Mean air temperature had a negative effect on the toothpick index and surface casting, while mean humidity had a positive effect on them (Table 1).

**Table 1:** Earthworm activity (measured by toothpick index and surface casting activity) in response to light pollution (LP), plant species (PS), sowing depth (SD), their interactions, and the covariates initial worm weight, soil moisture, and humidity. Significance code for Pr (> ChiSq): *** <0.001, ** <0.01, * <0.05

Earthworm surface activity				
	Toothpick index		Surface casting activity	
Parameters	Df	Pr (> ChiSq)	Df	Pr (> ChiSq)
Light pollution (LP)	1	< 2.200e^− 16^***	1	1.410e^− 05^***
Plant species (PS)	1	0.604	1	0.990
Sowing depth (SD)	1	0.919	1	0.060
LP x PS	1	0.037*	1	0.090
LP × SD	1	4.139e^− 04^***	1	0.007**
PS × SD	1	0.227	1	0.318
LP × SD × PS	1	0.001**	1	0.755
Initial worm weight (g)	1	0.901	1	0.567
Soil moisture (%)	1	0.503	1	0.591
Air humidity (%)	1	0.009**	1	5.223e^− 05^***
Air temperature (°C)	1	1.350e^− 11^***	1	3.201e^− 08^***

The number of earthworms was reduced by 27% at the end of the experiment and significantly correlated with light pollution and soil moisture content, which were measured at the end of the experiment (Table 2). Earthworms body weight was 3.4 ± 17.5% higher at the end of the experiment than at the beginning. None of the experimental factors had a significant effect on body weight, but the covariate of initial earthworm weight did: Initially heavier earthworms lost weight, while initially lighter earthworms gained weight over the course of the experiment.

**Fig. 2:**
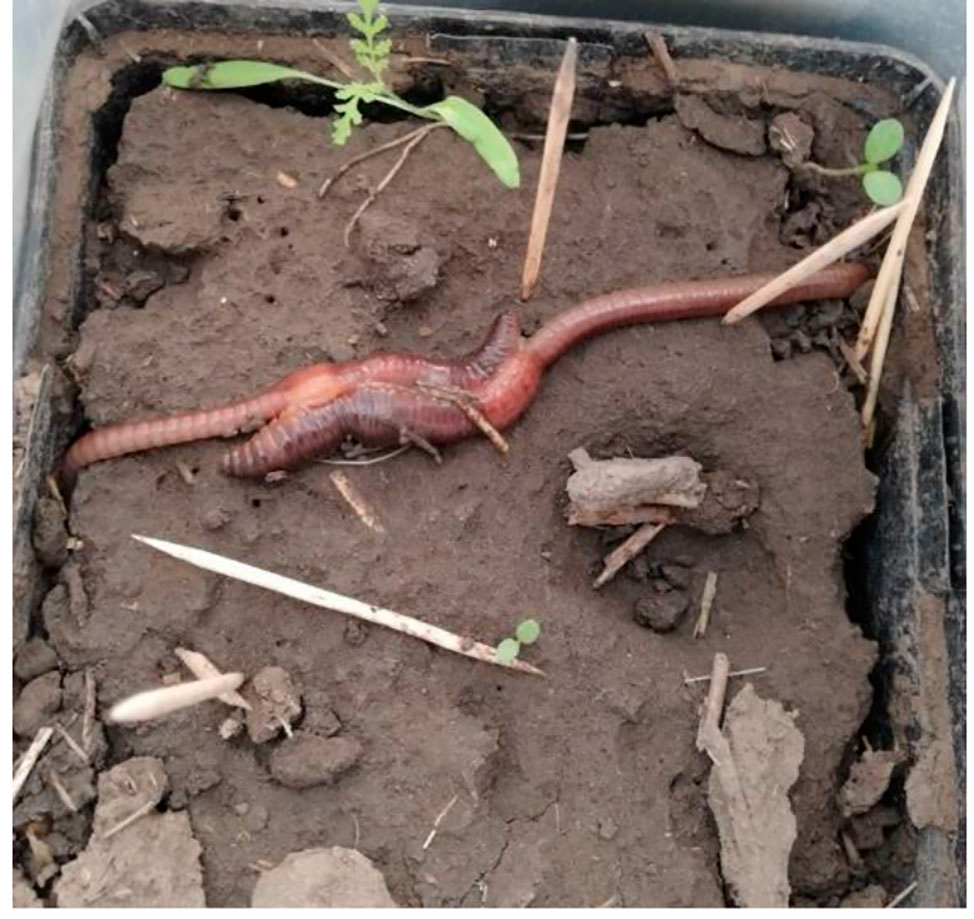
Two *L. terrestris* individuals during mating. Also seen are toothpicks used to determine surface activity and germinated seedlings of ragweed (top right and bottom centre) and *Phacelia* (top centre)

Mating behaviour was observed on seven occasions, six of which occurred in the dark treatment (Fig. 2).

**Table 2:** Change in earthworm numbers and biomass from start to end of the experiment in response to light pollution (LP), plant species (PS), sowing depth (SD), their interactions, and the covariates initial worm weight and soil moisture. ChiSq = Likelihood ratio Chi squared, df = degrees of freedom, Significance code for Pr(> ChiSq) and Pr(> F): *** <0.001, ** <0.01, * <0.05

	Earthworm number change		Earthworm biomass change	
Parameters	Df	Pr (> ChiSq)	Df	Pr (> F)
Light pollution (LP)	1	0.015*	1	0.169
Plant species (PS)	1	0.573	1	0.097
Sowing depth (SD)	1	0.220	1	0.305
LP x PS	1	0.474	1	0.371
LP × SD	1	0.891	1	0.429
PS × SD	1	0.198	1	0.069
LP × SD × PS	1	0.054	1	0.437
Initial worm weight (g)	1	0.118	1	0.011*
Soil moisture (%)	1	0.056	1	0.547
Residuals			33	

### Ragweed germination and growth

**Fig. 3:**
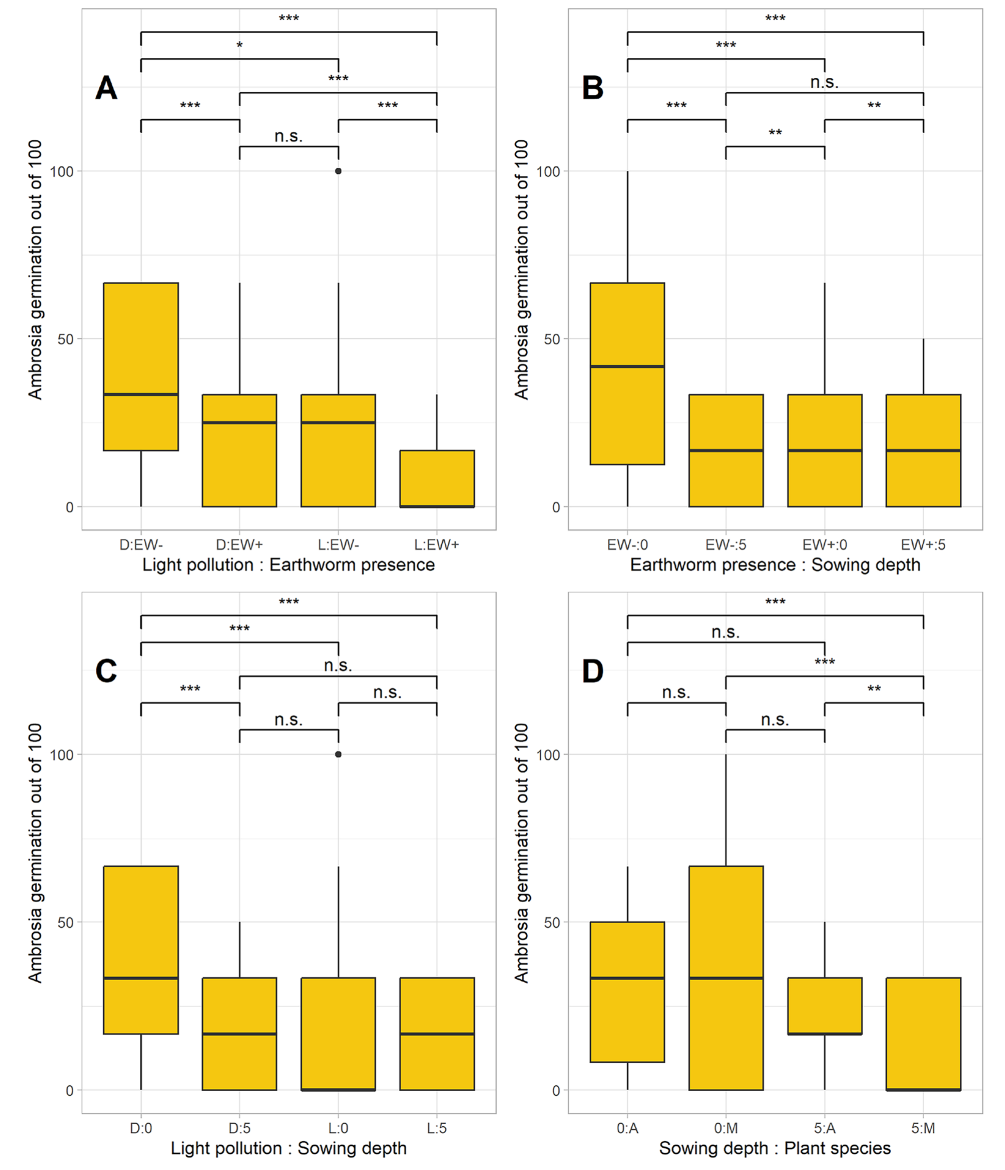
Ragweed germination of 100 in response to experimental factors light pollution (**A**, **C**), earthworms (**A**, **B**), sowing depth (**B, C, D**), and plant species (**D**). Abbreviations: Light pollution: D…dark, L…light; Earthworms: EW+…present, EW-…absent; Sowing depth: 0…sown at sown, 5…sown in 5 cm depth; Plant species: A…only Ragweed sown, M…Ragweed seeds and *Phacelia* seeds sown. *N* = 6

Overall, 24% of all ragweed seeds germinated, with 89% of these seedlings surviving to the end of the experiment. Ragweed germination was significantly affected by all experimental factors (Table 3; Fig. 3) and many of their interactions. Among the main effects, seed germination was reduced by 42% by earthworms, 33% by light pollution, 39% when seeds were buried, and 7% in seed mixes with *Phacelia*.

Ragweed germination was reduced when earthworms were present (Fig. 3A, B), and under light pollution (Fig. 3A, C). Interactions between the factors earthworms and light pollution resulted in the lowest germination success under light pollution with earthworms present (Fig. 3A). However, earthworms primarily affected surface sown seeds, while buried seeds germinated similarly, regardless of the presence of earthworm (Fig. 3B). Light pollution also had a stronger effect on surface-sown seeds, while buried seeds did not differ significantly in their response to light pollution or darkness (Fig. 3C).

Sowing depth interacted with plant species, with germination success being higher for surface sown seeds, while germination was similar for pure ragweed seeds and mixed seeding that also contained *Phacelia*. For buried seeds, ragweed germination was lower in the mixed treatment (Fig. 3D). Most of the two-way and three-way interactions had a significant effect on ragweed germination (Table 3).

**Fig. 4:**
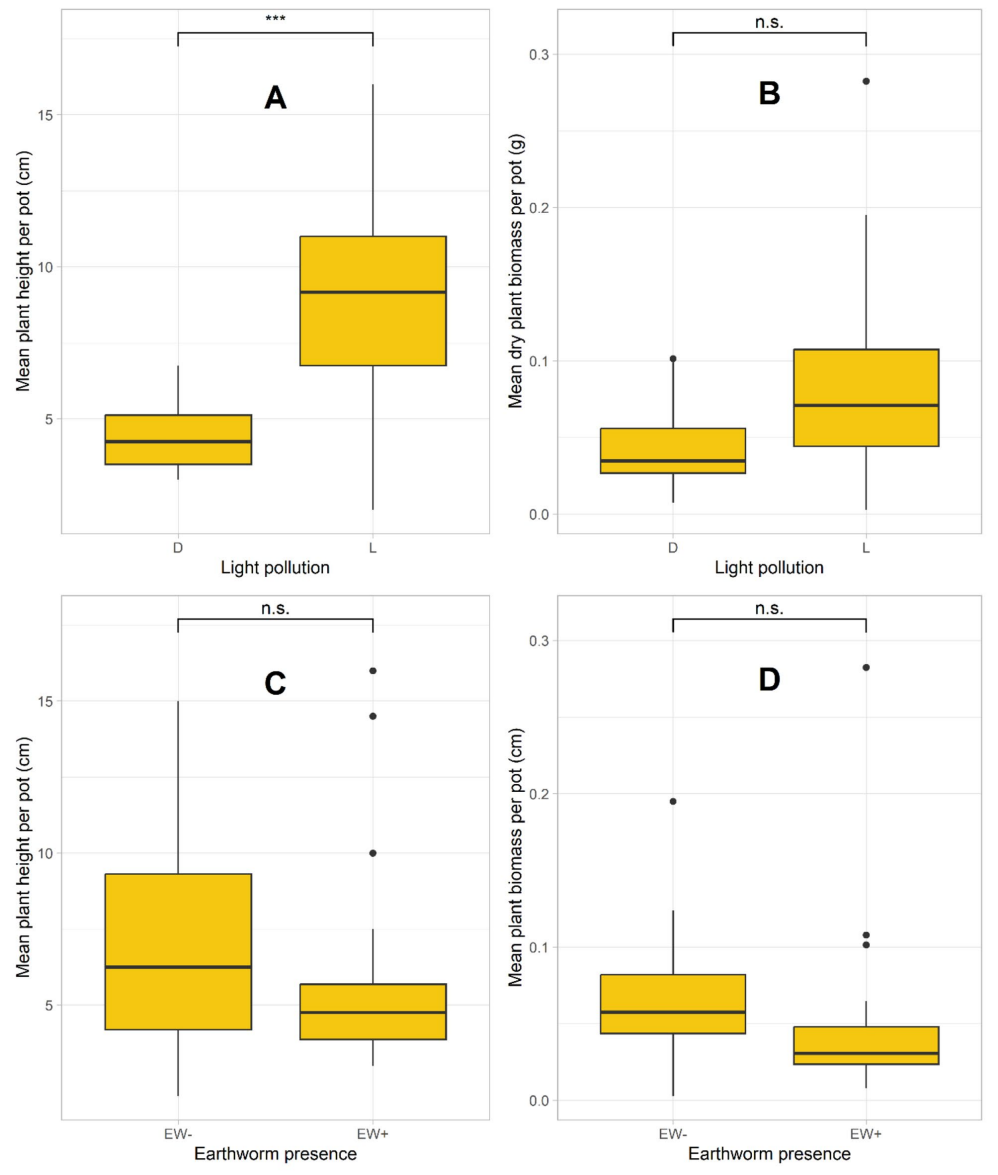
Mean ragweed plant height and plant biomass in response to light pollution (**A, B**) and earthworm presence (**C, D**). Abbreviations: D…dark, L…light, EW-…Earthworms absent, EW+…Earthworms present, n.s….not significant, *** *p*-value < 0.001, ** *p*-value < 0.01, * *p*-value < 0.05. *N* = 6

Plant growth was significantly affected by light pollution (Table 3), as plants grew taller when exposed to light at night (Fig. 4A); no other factor had a significant effect on plant height (Table 3). Plant biomass was marginally higher under light pollution (Fig. 4B) and when earthworms were present (Fig. 4C, D). Two-way and three-way interactions had no influence on ragweed height growth and biomass production (Table 3).

**Table 3:** Ragweed germination, mean plant biomass, and mean plant height in response to light pollution (LP), earthworms (EW), plant species (PS), sowing depth (SD) and their interactions. Mean plant height and biomass analyses only considering the 56 pots containing ragweed plants at the end. Pr (> ChiSq) and Pr (> F) significance codes: *** <0.001, ** <0.01, * <0.05

	Germination		Mean plant biomass		Mean plant height	
Parameters	Df	Pr (> ChiSq)	Df	Pr (> F)	Df	Pr (> F)
Light pollution (LP)	1	< 2.200e^− 16^***	1	0.054	1	3.490e^− 07^***
Earthworms (EW)	1	< 2.200e^− 16^***	1	0.068	1	0.208
Plant species (PS)	1	0.044*	1	0.662	1	0.618
Sowing depth (SD)	1	< 2.200e^− 16^***	1	0.857	1	0.140
LP x EW	1	1.508e^− 09^***	1	0.970	1	0.699
LP × SD	1	5.730e^− 14^***	1	0.288	1	0.479
LP x PS	1	0.236	1	0.686	1	0.443
EW × SD	1	< 2.200e^− 16^***	1	0.578	1	0.983
EW x PS	1	0.102	1	0.740	1	0.540
SD × PS	1	2.999e^− 05^***	1	0.808	1	0.393
LP x EW × SD	1	4.575e^− 06^***	1	0.990	1	0.862
LP × SD × PS	1	0.002**	1	0.253	1	0.600
EW × SD × PS	1	0.010*	1	0.717	1	0.934
LP x EW x PS	1	0.063	1	0.751	1	0.802
Residuals			41		41	

For the dark treatment, it should be noted that the darkness lasted 12 h and was therefore longer than a natural night in spring when the experiment was carried out. For the light treatment, natural daylight of more than 12 h led to comparatively shorter nights.

All raw data are provided in the supplementary material (Supplementary Table S1, Table S2, Table S3).

## Discussion

### Earthworm activity

In this study, it was shown that artificial light at night, as emitted by street lights or even skyglow of urban agglomerations, reduces the surface activity of the anecics earthworm *Lumbricus terrestris* which forages and mates on the soil surface during the night. The effects of light pollution on earthworms have so far only been reported in one other study so far [24]. These effects on earthworm activity may also affect the ecosystem services that earthworms provide, such as an improved nutrient cycling, or better soil aeration and water infiltration [32, 45]. Earthworm activity often leads to an increase in plant biomass production [32, 33]. In contrast, we found a marginally significant (*p* = 0.0681) decrease in ragweed biomass due to earthworm activity, suggesting that ragweed did not benefit from the earthworm-induced improvements in nutrients and soil structure, or that earthworms might have fed on roots as because they were foraging less on the surface [46] Our results show that the positive influence of earthworms on plant growth and soil properties can be reduced by light pollution.

Light pollution can be a particular problem for anecics earthworms, as their nocturnal foraging and mating takes place at the surface [28, 29]. Indeed, 86% of the earthworm matings observed in this study occurred in absence of light pollution, suggesting that light pollution provided less favourable conditions for mating. Similarly, earthworm losses were higher with light pollution (35% decrease in earthworm numbers during light pollution vs. 19% in the absence of light pollution), also indicating less favourable conditions. However, light pollution could also have a positive effect on earthworm populations, as they are less likely to be preyed upon by birds, amphibians, or hedgehogs [47] if they spend less time on the soil surface due to light pollution. Several studies have found that light pollution can affect the relationship between predators and prey [13, 48], with prey showing increased vigilance towards predators in the presence of light pollution [16], or avoiding illuminated areas when foraging [17], but none of these studies examined the situation of earthworms. Predator-prey interactions are complex, as animal species can be both predators and prey. However, at the community levels predator species tend to thrive better under light pollution than others [49, 50]. In addition, there is evidence that animal species that are normally only active during the day remain active after sunset due to light pollution, such as birds that prey on earthworms [51, 52]. Thus, earthworms may be exposed to increased predation pressure due to light pollution, which, in addition to reducing mating and foraging due to lower surface activity, puts pressure on earthworm populations as a whole.

The activity of earthworms was also influenced by the sowing depth with a higher activity when seeds were surface sown, especially in the absence of light pollution. This suggests that earthworms may have fed on these seeds [35, 36, 53]. Furthermore, the seeds were not the only food on the soil surface that the earthworms may have foraged for, as they were also repeatedly fed with shredded hay. However, earthworm activity was increased when they also had easy access to the seeds rich in protein and fat [54]. We could not observe an interaction between earthworms and buried seeds, as the germination success of buried seeds did not differ whether earthworms were present or not. There is also evidence that earthworms interact with buried seeds by moving the seeds vertically in the soil [36], which could not be confirmed in this study.

### Ragweed germination and growth

The overall low germination rate of 24% for ragweed was consistent with reported germination rates of up to 25% [55, 56]. However, much higher germination rates were obtained under ideal laboratory conditions [55, 57]. Earthworms decreased germination rates of *Ambrosia artemisifolia*, confirming the observations of a study [37, 55]in which earthworms reduced seedling emergence of the related noxious annual weed giant ragweed (*Ambrosia trifida*) [35]. Earthworms significantly interacted with light pollution: Germination was generally reduced by light pollution, but earthworms further enhanced the reducing effect of light pollution. It has been shown that ragweed germination is most successful with alternating light and dark periods [57]. In this study, a complete alternation between light and dark was observed in the absence of light pollution, which explains the slightly higher germination rate in this treatment.

The germination of ragweed was less successful with buried seeds, which confirms previous studies [57, 58]. The sowing depth of 5 cm, as used in this experiment, thus led to an expected reduction in seedling emergence, but not to a complete absence in emergence. In the field, the seeds are often buried by tillage, which leads to a lower germination rate in the short-term. In the long-term, however, the seeds are added to the soil seed bank, from which they can germinate when they are brought closer to the soil surface again. For example, earthworms have been shown to bury seeds [35] or move them from the soil seed bank to the soil surface [36].

Despite lower ragweed germination under light pollution, the plant height growth was increased. This suggests that light pollution provides additional resources for photosynthesis leading to a growth advantage [11]. At the same time, no other factor affected ragweed growth, not even the presence of earthworms, which was unexpected as earthworms have been shown to increase plant biomass production even if they decrease germination rate [35, 53]. However, an increase in biomass by earthworms was not demonstrated in this experiment, which may be due to the relatively short duration of the experiment. For example, the growth-promoting effect of earthworm casts depends on their age, with fresh casts having less effect on plant growth than older ones [59].

## Conclusion

This is one of the first studies to demonstrate the effects of light pollution on interactions between earthworms and the invasive neophyte ragweed. This is particularly important as both light pollution [60] and ragweed infestation [61] are expected to increase in the coming years. In this greenhouse experiment, we showed that moderate light pollution, such as found in home gardens or near streetlights, reduces the surface activity of anecics earthworms and increases the growth of ragweed. The extent to which this affects the life history and fitness of earthworms and ragweed under field conditions is unclear. In any case, the impact of light pollution on earthworms can have far-reaching consequences, both for earthworm populations and for organisms who benefit from the many ecosystem services they provide. As earthworms are strongly involved in complex ecological interactions in ecosystems [62, 63] and many other organisms could be affected by light pollution at the same time [64], this could have many ramifications. If increased ragweed growth under light pollution would lead to higher pollen and seed production [65], this could contribute to a further increase in ragweed infestation and even to an increased risk of ragweed pollen allergy [43]. However, these complex relationships need to be further investigated.

## Methods

### Experimental setup

A full factorial experiment was conducted in the research greenhouse of the University of Natural Resources and Life Sciences Vienna (BOKU) in April and May 2022 (45 days).

Four factors with two levels each were considered:

- Factor light pollution (LP): complete darkness (D) vs. artificial light pollution (L).
- Factor earthworms (EW): *L. terrestris* present (EW+) vs. absent (EW-).
- Factor plant species (PS): seeding *A. artemisiifolia* alone (A) vs. in combination with *Phacelia tanacetifolia* (M).
- Factor sowing depth (SD): surface sown seeds (0) vs. sowing depth of 5 cm (5).

This resulted in (2 x LP) x (2 x EW) x (2 x PS) x (2 × SD) x 6 replicates = 96 experimental units.

The experimental units consisted of plastic plant pots (15 × 15 × 20 cm, L x W x H) with a volume of three litres. To prevent earthworms from escaping, the bottoms of the pots were taped shut with mosquito netting to cover drainage holes. Additionally, a vertical clear plastic barrier of 15 cm height was taped to the rim of all pots; also, the rim of the plastic barrier was smeared with soft soap on the inside to further deter earthworms from escaping. The soil that was used was topsoil (0 to 15 cm) from an organic arable field of the BOKU Experimental Farm in Groß-Enzersdorf near Vienna with the following characteristics as analysed by the Austrian Agency for Health and Food Safety (AGES): pH (CaCl_2_) = 7.6, *P* = 73 mg kg^− 1^, K = 167 mg kg^− 1^, soil organic matter = 3.9%. The soil was sieved (mesh size 1 cm) and added to the pots in equal amounts, with a bulk density of 1 g cm^− 3^.

The pots were arranged on two greenhouse tables and randomized once in the beginning of the experiment. Additionally, the position of the tables, in the greenhouse cabinet was changed after four weeks of the experiment.

**Fig. 5:**
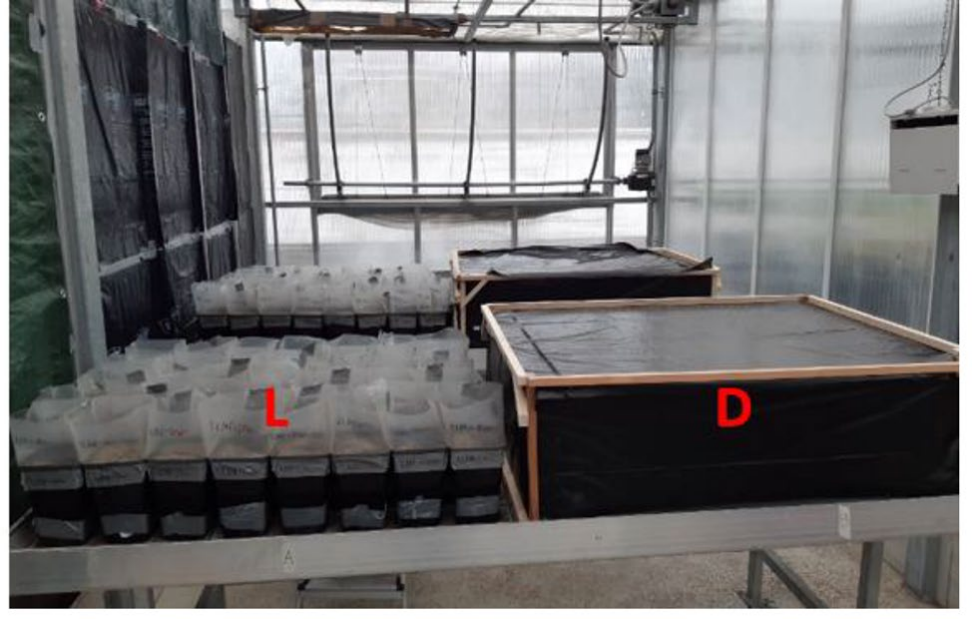
Experimental setup in the greenhouse. Light pollution (L) was achieved by covered ceiling lights, and the dark (D) treatment by covering pots with opaque plastic sheets

For the factor light pollution, level “light” was achieved by keeping half of the pots under artificial light pollution every night between 8 p.m. and 8 a.m. in the following morning. Therefore, the existing ceiling lights (fluorescent tubes, 36 W, 4000 K) were switched on overnight, but covered with a thin layer of dark plastic foil. Factor level “dark” was achieved by keeping the other half of the pots in complete darkness every night from 8 p.m. to 8 a.m. For this purpose, a wooden frame lined with black, opaque plastic foil was placed over the pots (Fig. 5).

For factor earthworms, the factor level ”including earthworms” was established by adding two adult individuals of *L. terrestris* to half of the pots. The earthworms were purchased from a fishing supply shop and stored in a climate chamber at 15 °C for one week prior to the start of the experiment. Then two individuals (8.9 ± 1.0 g pot^− 1^) were washed, weighed and added to the pots. This corresponds to an earthworm biomass of 395 g m^− 2^, which would be the cumulative weight of 88 individuals m^− 2^. This population density is plausible for an arable field, although higher densities can also occur in the field [62]. Pots with factor level “excluding earthworms” did not receive any earthworms.

Factor plant species was established by sowing six seeds per pot in a 2 × 3 matrix using a template to ensure equal placement. For factor level “Ambrosia”, only ragweed seeds were added, while pots with factor level “Mixed seeds” received a combination of ragweed and *Phacelia* seeds, three seeds each, placed alternately in the matrix. This corresponds to a sowing density of 266 seeds m^− 2^. After the seeds were placed, 250 ml of water were added to each pot. The seeds of *A. artemisiifolia* were obtained from the Institute of Botany, University of Natural Resources and Life Sciences, Vienna; seeds of *P. tanacetifolia* were obtained from the BOKU Research Farm in Groß-Enzersdorf, near Vienna, Austria.

Factor sowing depth was established by either placing the seeds on the soil surface or at a depth of 5 cm.

The pots were watered evenly with 150 to 200 ml pot^− 1^ every three to four days during the first week, which was then reduced to 100 ml pot^− 1^ for the rest of the experiment. In addition, hay cut into small pieces (< 1 cm) was added as earthworm feed. This was done on average once per week throughout the experiment, with 1 g always added to each pot. For the first three weeks, hay was added to all pots, after which the application was reduced to pots including earthworms so as not to impede plant growth by accumulating hay biomass on the surface of pots without any earthworms. Unwanted germinating seedlings were removed as soon as they could be identified as such. Dead earthworms that were on the surface were also removed.

The average air temperature for the duration of the experiment was 21.2 ± 1.5 °C with an average air humidity of 61.7 ± 13.6% and 72.7 ± 10.8% in “light” and “dark” treatments respectively.

### Measurements

**Fig. 6:**
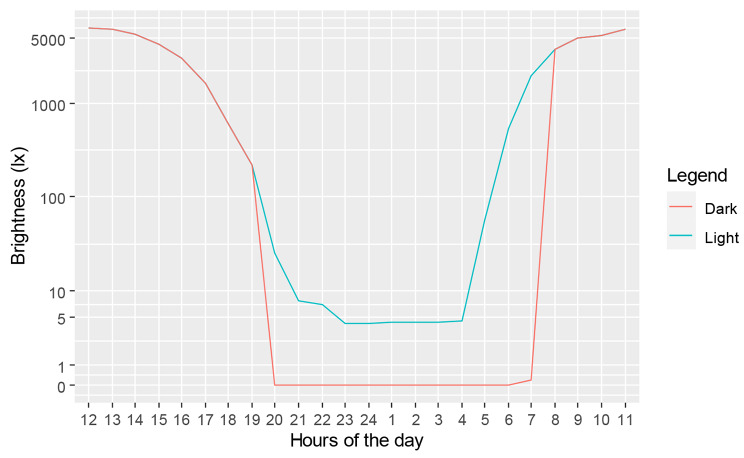
Mean brightness measured throughout all experimental days comparing the dark (D) and light (L) treatments

Light levels were measured at 30-minute intervals throughout the experiment using a luxmeter (Voltcraft LX-2000, Conrad Electronic SE, Hirschau, Germany). The luxmeter was placed on the soil surface of an extra pot under the same conditions as the experimental pots. Since only one luxmeter was available, it was moved between “light” and “dark” treatments, and between different areas of the experimental pots. A total of 29 nights were recorded, with the luxmeter placed in the “light” treatment 72% of the time and in the “dark” treatment 28% of the time. Based on these measurements, the average light level from 9 pm to 4 am in the “light” treatment was 4.3 ± 1.5 lx, which is about 14 times brighter than the full moon [66]. Between 8 pm and 8 am, it was 218 ± 129 lx due to early sunrise at the end of the experiment. The average light level from 8 pm to 8 am in the “dark” treatment was 0 lx (Fig. 6).

Earthworm surface activity was assessed only in pots containing earthworms using the toothpick method, in which the number of toothpicks moved serves as a measure of earthworm activity [56]. Therefore, six wooden toothpicks pot^− 1^ were inserted vertically into the soil surface in the evening, penetrating only 2–3 mm into the soil. When earthworms come to the surface at night to forage, they drop the toothpicks or move them in an inclined position. In the next morning, the number of inclined or fallen toothpicks were counted: One point was assigned to each toothpick that fell over, 0.5 points were assigned to each inclined toothpick, the sum of which was the toothpick index. In addition, earthworm activity was recorded based on the number of earthworm surface casts. Casts are stable structures mostly consisting of soil, which are excreted by earthworms as faeces, and their number and weight can be used as a measure of earthworm activity [62]. After each assessment the casts were crumbled onto the soil surface to avoid assessing the same casts in the future. A total of 12 earthworm activity assessments were made throughout the experiment, with an average of two assessments per week. Earthworm biomass was determined by weighing the worms at the beginning and end of the experiment, and the worms were always washed and blotted dry with a paper towel beforehand.

Ragweed germination was assessed by repeatedly counting all seedlings that germinated throughout the experiment. *Phacelia* germination was not further considered in this study.

Plant growth was determined by measuring the plant height four times during the experiment from the soil surface to the highest central nodule of each plant. The plant biomass pot^− 1^ was determined at the end of the experiment by cutting the plants at the soil surface. Plant material was dried at 55 °C for 48 h and weighed. At the end of the experiment, the pots were flipped over, earthworms were sorted out, and soil samples were collected.

Air temperature and humidity were recorded continuously with eight tinytag data loggers (Gemini Data Loggers, Chichester, UK) evenly distributed between “light” and “dark” treatments. Soil temperature and moisture were recorded only once at the end of the experiment. Soil temperature was measured using a digital thermometer with a metal probe inserted 15 cm deep into the centre of the soil surface. Soil moisture content was determined by taking a soil core from the centre of each pot using a 20 ml plastic syringe (diameter 2 cm), with the tip cut off. The soil core was then weighed, dried at 100 °C for 48 h, and weighed again to calculate the gravimetric water content.

### Statistical analysis

Statistical analysis was performed using R version 4.3.1 [67] with a significance level of 5% (α = 0.05) [66]. 

Five pots from different treatments were excluded because no live earthworms could be recovered at the end of the experiment. This results in a lower number of replicates for some factor combinations, namely four replicates in two cases and five replicates in one case.

As for earthworm activity, toothpick index and surface casting activity were analysed using Generalized Linear Mixed Models (GLMMs) to account for repeated measures. The R package glmmTMB [68] with a Poisson distribution was used for toothpick index analysis. A binomial distribution was chosen for surface casting activity after recoding the surface cast counts to a presence/absence format. The experimental factors were used as fixed effect, while the individual pot ID was added as a random effect to account for dependencies between repeated measurements recorded within the same pots.

Earthworm losses and ragweed germination were analysed using Generalized Linear Models (GLMs), while the plant biomass and plant height were analysed using a multifactorial ANOVA based on log-transformed data to ensure normality.

In the earthworm analyses, several covariates were also included in the models. This was done because earthworms are relatively sensitive to environmental conditions, such as temperature and humidity, which can influence their activity levels [62]. Therefore, mean air temperature and mean air humidity were included as covariates in all repeated measures models, as the temperature and humidity values of the individual sampling days could be paired with the corresponding measurements. Further, earthworm weight at the beginning of the experiment and soil moisture at the end of the experiment were included for all earthworm analyses. Analyses of ragweed data did not include any covariates.

All graphs were generated using the packages ggplot2 [69] and ggpubr [70]. Post-hoc group-wise comparisons of means for GLM(M)s were performed using the package emmeans [71], with comparison results being added to graphs using the package ggsignif [72].

### Electronic Supplementary Material

Below is the link to the electronic supplementary material.

**Supplementary Material 1:** Supplementary Tables

## Data Availability

All data generated or analyzed during this study are included in this published article.
