# Supplementary material for: Artificial light at night reduces earthworm activity but increases growth of invasive ragweed
Source: BMC Ecol Evol. 2024 Jan 19;24:10. doi: 10.1186/s12862-024-02200-x (PMC10797752; doi:10.1186/s12862-024-02200-x)
Supplement: Supplementary file 1 — Supplementary Material 1: Supplementary Tables [file 12862_2024_2200_MOESM1_ESM.docx]

# Supplementary material

**Supplementary Table S1** Raw data on effects of light pollution on earthworm weight and ragweed germination and growth. Abbreviations: LP…light pollution, SD…sowing depth, PS…plant species, EW…earthworms.

| **Pot code** | **LP** | **SD** | **PS** | **EW** | **Initial worm weight mean (g)** | **Final worm weight mean (g)** | **Worm loss (%)** | **Ambrosia germi-nation rate (%)** | **Mean plant height (cm)** | **Mean plant biomass (g)** | **Soil moisture content (%)** |
| --- | --- | --- | --- | --- | --- | --- | --- | --- | --- | --- | --- |
| 1D0AEW+ | D | 0 | A | EW+ | 4.23 | 4.67 | 50% | 16.7% | 4 | 0.0375 | 31.4% |
| 2D0AEW+ | D | 0 | A | EW+ | 5.35 | 6.08 | 50% | 50.0% | 5.25 | 0.0322 | 35.6% |
| 3D0AEW+ | D | 0 | A | EW+ | 4.21 | 4.805 | 0% | 16.7% | 5 | 0.0317 | 36.4% |
| 4D0AEW+ | D | 0 | A | EW+ | 4.26 | 4.42 | 0% | 0.0% |  |  | 24.3% |
| 5D0AEW+ | D | 0 | A | EW+ | 4.48 | 4.715 | 0% | 33.3% | 3 | 0.0111 | 24.8% |
| 6D0AEW+ | D | 0 | A | EW+ | 4.72 | 4.71 | 0% | 0.0% |  |  | 30.6% |
| 1D5AEW+ | D | 5 | A | EW+ | 4.54 | 4.855 | 0% | 16.7% | 3 | 0.0253 | 32.4% |
| 2D5AEW+ | D | 5 | A | EW+ | 4.76 | 4.28 | 0% | 33.3% | 6.25 | 0.1014 | 33.1% |
| 3D5AEW+ | D | 5 | A | EW+ | 4.73 | 5.385 | 0% | 50.0% | 3.25 | 0.0171 | 30.1% |
| 4D5AEW+ | D | 5 | A | EW+ | 3.50 | 3.99 | 0% | 16.7% | 4.5 | 0.0572 | 28.7% |
| 5D5AEW+ | D | 5 | A | EW+ | 3.70 | 4.37 | 50% | 33.3% | 3.25 | 0.0275 | 29.2% |
| 6D5AEW+ | D | 5 | A | EW+ | 3.87 | 4.035 | 0% | 0.0% |  |  | 28.0% |
| 1D0AEW- | D | 0 | A | EW- |  |  |  | 66.7% | 5.625 | 0.065325 | 25.2% |
| 2D0AEW- | D | 0 | A | EW- |  |  |  | 66.7% | 6.75 | 0.079825 | 26.4% |
| 3D0AEW- | D | 0 | A | EW- |  |  |  | 0.0% |  |  | 23.6% |
| 4D0AEW- | D | 0 | A | EW- |  |  |  | 66.7% | 3 | 0.0446 | 25.0% |
| 5D0AEW- | D | 0 | A | EW- |  |  |  | 66.7% | 4.25 | 0.0073 | 26.9% |
| 6D0AEW- | D | 0 | A | EW- |  |  |  | 50.0% | 5.833 | 0.05533 | 24.2% |
| 1D5AEW- | D | 5 | A | EW- |  |  |  | 33.3% | 4.25 | 0.05635 | 31.6% |
| 2D5AEW- | D | 5 | A | EW- |  |  |  | 16.7% | 5 | 0.099 | 22.3% |
| 3D5AEW- | D | 5 | A | EW- |  |  |  | 16.7% | 4 | 0.0584 | 31.8% |
| 4D5AEW- | D | 5 | A | EW- |  |  |  | 16.7% |  |  | 28.8% |
| 5D5AEW- | D | 5 | A | EW- |  |  |  | 16.7% | 3.5 | 0.0421 | 27.9% |
| 6D5AEW- | D | 5 | A | EW- |  |  |  | 16.7% |  |  | 25.3% |
| 1D0MEW+ | D | 0 | M | EW+ | 4.71 | 5.355 | 0% | 66.7% | 4.25 | 0.02945 | 26.7% |
| 2D0MEW+ | D | 0 | M | EW+ | 4.12 | 5.485 | 0% | 0.0% |  |  | 31.7% |
| 3D0MEW+ | D | 0 | M | EW+ | 4.59 | 4.89 | 0% | 33.3% | 3.5 | 0.0139 | 27.8% |
| 4D0MEW+ | D | 0 | M | EW+ | 4.23 | 4.78 | 0% | 33.3% | 4 | 0.0258 | 31.9% |
| 5D0MEW+ | D | 0 | M | EW+ | 4.45 | 4.43 | 0% | 33.3% | 5 | 0.0346 | 28.3% |
| 6D0MEW+ | D | 0 | M | EW+ | 4.47 | 5.185 | 0% | 33.3% | 5 | 0.0261 | 28.2% |
| 1D5MEW+ | D | 5 | M | EW+ | 4.84 | 5.02 | 50% | 0.0% |  |  | 30.5% |
| 2D5MEW+ | D | 5 | M | EW+ | 4.34 | 0 | 100% | 33.3% | 4 | 0.0806 | 30.7% |
| 3D5MEW+ | D | 5 | M | EW+ | 4.83 | 3.96 | 0% | 0.0% |  |  | 27.8% |
| 4D5MEW+ | D | 5 | M | EW+ | 5.01 | 3.685 | 0% | 0.0% |  |  | 30.2% |
| 5D5MEW+ | D | 5 | M | EW+ | 4.58 | 5.14 | 50% | 33.3% | 4 | 0.029 | 22.8% |
| 6D5MEW+ | D | 5 | M | EW+ | 3.71 | 0 | 100% | 0.0% |  |  | 28.1% |
| 1D0MEW- | D | 0 | M | EW- |  |  |  | 66.7% | 6.25 | 0.0757 | 26.8% |
| 2D0MEW- | D | 0 | M | EW- |  |  |  | 66.7% | 6 | 0.04925 | 25.2% |
| 3D0MEW- | D | 0 | M | EW- |  |  |  | 33.3% |  |  | 27.3% |
| 4D0MEW- | D | 0 | M | EW- |  |  |  | 66.7% | 3.5 | 0.0331 | 19.2% |
| 5D0MEW- | D | 0 | M | EW- |  |  |  | 33.3% | 3 | 0.0457 | 28.8% |
| 6D0MEW- | D | 0 | M | EW- |  |  |  | 0.0% |  |  | 29.9% |
| 1D5MEW- | D | 5 | M | EW- |  |  |  | 33.3% | 4 | 0.0407 | 28.3% |
| 2D5MEW- | D | 5 | M | EW- |  |  |  | 0.0% |  |  | 26.8% |
| 3D5MEW- | D | 5 | M | EW- |  |  |  | 33.3% | 3.5 | 0.0285 | 26.2% |
| 4D5MEW- | D | 5 | M | EW- |  |  |  | 33.3% | 6 | 0.0253 | 26.1% |
| 5D5MEW- | D | 5 | M | EW- |  |  |  | 0.0% |  |  | 30.6% |
| 6D5MEW- | D | 5 | M | EW- |  |  |  | 0.0% |  |  | 28.2% |
| 1L0AEW+ | L | 0 | A | EW+ | 4.38 | 0 | 100% | 0.0% |  |  | 29.9% |
| 2L0AEW+ | L | 0 | A | EW+ | 4.58 | 4.63 | 50% | 16.7% | 7 | 0.045 | 25.4% |
| 3L0AEW+ | L | 0 | A | EW+ | 4.91 | 4.865 | 0% | 16.7% | 16 | 0.2823 | 22.7% |
| 4L0AEW+ | L | 0 | A | EW+ | 4.77 | 5.135 | 0% | 0.0% |  |  | 35.2% |
| 5L0AEW+ | L | 0 | A | EW+ | 3.63 | 3.47 | 50% | 33.3% | 5 | 0.00775 | 33.2% |
| 6L0AEW+ | L | 0 | A | EW+ | 4.23 | 4.215 | 0% | 0.0% |  |  | 32.4% |
| 1L5AEW+ | L | 5 | A | EW+ | 5.05 | 2.79 | 50% | 16.7% | 10 | 0.1079 | 32.4% |
| 2L5AEW+ | L | 5 | A | EW+ | 5.04 | 4.1 | 50% | 16.7% | 4.5 | 0.0646 | 25.9% |
| 3L5AEW+ | L | 5 | A | EW+ | 5.06 | 6.05 | 50% | 16.7% | 7.5 | 0.0183 | 27.9% |
| 4L5AEW+ | L | 5 | A | EW+ | 3.50 | 5.055 | 0% | 0.0% |  |  | 28.3% |
| 5L5AEW+ | L | 5 | A | EW+ | 3.70 | 5.19 | 50% | 16.7% | 3.5 | 0.0162 | 29.5% |
| 6L5AEW+ | L | 5 | A | EW+ | 3.87 | 4.74 | 50% | 0.0% |  |  | 28.1% |
| 1L0AEW- | L | 0 | A | EW- |  |  |  | 33.3% | 9.5 | 0.1066 | 23.9% |
| 2L0AEW- | L | 0 | A | EW- |  |  |  | 50.0% | 9 | 0.07086 | 29.0% |
| 3L0AEW- | L | 0 | A | EW- |  |  |  | 0.0% |  |  | 23.9% |
| 4L0AEW- | L | 0 | A | EW- |  |  |  | 16.7% | 11 | 0.1182 | 29.6% |
| 5L0AEW- | L | 0 | A | EW- |  |  |  | 33.3% | 11.5 | 0.07595 | 27.3% |
| 6L0AEW- | L | 0 | A | EW- |  |  |  | 33.3% | 11 | 0.11885 | 29.0% |
| 1L5AEW- | L | 5 | A | EW- |  |  |  | 33.3% | 9.25 | 0.08535 | 25.7% |
| 2L5AEW- | L | 5 | A | EW- |  |  |  | 33.3% | 6.75 | 0.05375 | 29.2% |
| 3L5AEW- | L | 5 | A | EW- |  |  |  | 16.7% | 13 | 0.1237 | 31.9% |
| 4L5AEW- | L | 5 | A | EW- |  |  |  | 33.3% | 6.25 | 0.05395 | 29.0% |
| 5L5AEW- | L | 5 | A | EW- |  |  |  | 16.7% | 15 | 0.195 | 31.8% |
| 6L5AEW- | L | 5 | A | EW- |  |  |  | 16.7% | 2 | 0.0026 | 32.8% |
| 1L0MEW+ | L | 0 | M | EW+ | 4.06 | 3.43 | 50% | 0.0% |  |  | 27.9% |
| 2L0MEW+ | L | 0 | M | EW+ | 4.47 | 4.505 | 0% | 0.0% |  |  | 30.5% |
| 3L0MEW+ | L | 0 | M | EW+ | 4.90 | 4.88 | 50% | 0.0% |  |  | 30.0% |
| 4L0MEW+ | L | 0 | M | EW+ | 4.87 | 4.13 | 0% | 0.0% |  |  | 32.8% |
| 5L0MEW+ | L | 0 | M | EW+ | 4.74 | 5.59 | 0% | 0.0% |  |  | 33.4% |
| 6L0MEW+ | L | 0 | M | EW+ | 4.90 | 5.58 | 0% | 0.0% |  |  | 31.2% |
| 1L5MEW+ | L | 5 | M | EW+ | 3.93 | 3.17 | 50% | 33.3% |  |  | 24.5% |
| 2L5MEW+ | L | 5 | M | EW+ | 4.54 | 3.34 | 50% | 33.3% | 5.5 | 0.0379 | 26.5% |
| 3L5MEW+ | L | 5 | M | EW+ | 5.02 | 4.18 | 0% | 33.3% | 14.5 | 0.0602 | 27.6% |
| 4L5MEW+ | L | 5 | M | EW+ | 5.45 | 5.025 | 0% | 0.0% |  |  | 28.1% |
| 5L5MEW+ | L | 5 | M | EW+ | 4.124 | 0 | 100% | 0.0% |  |  | 25.9% |
| 6L5MEW+ | L | 5 | M | EW+ | 4.94 | 0 | 100% | 33.3% | 5 | 0.0511 | 26.7% |
| 1L0MEW- | L | 0 | M | EW- |  |  |  | 0.0% |  |  | 29.4% |
| 2L0MEW- | L | 0 | M | EW- |  |  |  | 0.0% |  |  | 23.3% |
| 3L0MEW- | L | 0 | M | EW- |  |  |  | 0.0% |  |  | 27.9% |
| 4L0MEW- | L | 0 | M | EW- |  |  |  | 66.7% | 9 | 0.07905 | 29.0% |
| 5L0MEW- | L | 0 | M | EW- |  |  |  | 100.0% | 9.166 | 0.04413 | 23.1% |
| 6L0MEW- | L | 0 | M | EW- |  |  |  | 66.7% | 10.75 | 0.0809 | 30.2% |
| 1L5MEW- | L | 5 | M | EW- |  |  |  | 33.3% | 7 | 0.0409 | 23.3% |
| 2L5MEW- | L | 5 | M | EW- |  |  |  | 0.0% |  |  | 27.9% |
| 3L5MEW- | L | 5 | M | EW- |  |  |  | 0.0% |  |  | 29.0% |
| 4L5MEW- | L | 5 | M | EW- |  |  |  | 0.0% |  |  | 23.1% |
| 5L5MEW- | L | 5 | M | EW- |  |  |  | 33.3% | 13 | 0.1073 | 30.2% |
| 6L5MEW- | L | 5 | M | EW- |  |  |  | 0.0% |  |  | 23.3% |

**Supplementary Table S2** Raw data on effects of light pollution on earthworms and ragweed. Earthworm activity assessed by toothpick index. Toothpick index on individual sampling days given in ordinal date. Abbreviations: LP…light pollution, SD…sowing depth, PS…plant species, EW…earthworms.

|  |  |  |  |  | **Sampling days** | | | | | | | | | | | |
| --- | --- | --- | --- | --- | --- | --- | --- | --- | --- | --- | --- | --- | --- | --- | --- | --- |
| **Pot code** | **LP** | **SD** | **PS** | **EW** | **103** | **105** | **108** | **110** | **113** | **116** | **122** | **126** | **133** | **134** | **137** | **141** |
| 1D0AEW+ | D | 0 | A | EW+ | 0 | 1 | 0 | 0.5 | 0 | 4 | 4 | 0.5 | 0 | 0 | 0 | 0 |
| 2D0AEW+ | D | 0 | A | EW+ | 0 | 3.5 | 0 | 3 | 0 | 2.5 | 0.5 | 0.5 | 0 | 0.5 | 1 | 0 |
| 3D0AEW+ | D | 0 | A | EW+ | 2 | 0.5 | 0.5 | 3 | 2.5 | 6 | 3 | 1 | 0 | 0 | 0 | 0.5 |
| 4D0AEW+ | D | 0 | A | EW+ | 0 | 0 | 1.5 | 1 | 1 | 5 | 5 | 1 | 0 | 0 | 3.5 | 3.5 |
| 5D0AEW+ | D | 0 | A | EW+ | 0.5 | 0 | 0 | 2.5 | 4.5 | 4 | 5.5 | 2 | 1.5 | 0 | 1 | 2 |
| 6D0AEW+ | D | 0 | A | EW+ | 0 | 1 | 0 | 0.5 | 2.5 | 4 | 3 | 0 | 0 | 0 | 0 | 1 |
| 1D5AEW+ | D | 5 | A | EW+ | 1.5 | 4 | 2 | 3 | 0.5 | 0 | 2.5 | 1 | 0 | 1 | 2.5 | 0 |
| 2D5AEW+ | D | 5 | A | EW+ | 0 | 3 | 3 | 1 | 2 | 5 | 6 | 4 | 0 | 0 | 0.5 | 1 |
| 3D5AEW+ | D | 5 | A | EW+ | 0 | 1.5 | 0 | 2 | 2.5 | 4 | 5.5 | 2 | 0 | 0 | 2.5 | 0 |
| 4D5AEW+ | D | 5 | A | EW+ | 0 | 0.5 | 0 | 2.5 | 5 | 4 | 6 | 2.5 | 0 | 0 | 2 | 1.5 |
| 5D5AEW+ | D | 5 | A | EW+ | 0 | 0.5 | 0 | 1.5 | 2.5 | 2 | 4 | 2 | 0 | 0 | 0 | 0 |
| 6D5AEW+ | D | 5 | A | EW+ | 0 | 0 | 2 | 1.5 | 0 | 2 | 0.5 | 0 | 0 | 1 | 0 | 0 |
| 1D0MEW+ | D | 0 | M | EW+ | 0 | 2 | 0 | 3.5 | 5 | 4.5 | 6 | 0 | 0 | 0 | 4 | 0 |
| 2D0MEW+ | D | 0 | M | EW+ | 0.5 | 1 | 0 | 2.5 | 3.5 | 5 | 5.5 | 1 | 0 | 1.5 | 3 | 0 |
| 3D0MEW+ | D | 0 | M | EW+ | 0 | 2 | 1 | 0 | 3.5 | 3 | 5.5 | 2 | 0 | 1 | 2.5 | 2 |
| 4D0MEW+ | D | 0 | M | EW+ | 0 | 0 | 1 | 1 | 3 | 5 | 5.5 | 2.5 | 0 | 0.5 | 3 | 1 |
| 5D0MEW+ | D | 0 | M | EW+ | 0 | 1 | 0 | 2.5 | 2.5 | 0.5 | 5 | 1 | 0 | 1 | 1 | 0 |
| 6D0MEW+ | D | 0 | M | EW+ | 0 | 1 | 0 | 3.5 | 5 | 3 | 6 | 1 | 0 | 0 | 0 | 2 |
| 1D5MEW+ | D | 5 | M | EW+ | 1 | 1 | 1 | 1.5 | 1.5 | 1 | 1 | 0 | 0 | 0 | 0 | 1 |
| 2D5MEW+ | D | 5 | M | EW+ | 0 | 1 | 0.5 | 1.5 | 1 | 1 | 3 | 0 | 0 | 0 | 0 | 0 |
| 3D5MEW+ | D | 5 | M | EW+ | 0 | 0 | 2 | 0.5 | 0.5 | 2 | 3.5 | 0 | 0 | 0.5 | 1.5 | 0 |
| 4D5MEW+ | D | 5 | M | EW+ | 0 | 0 | 0 | 0.5 | 2 | 1 | 2 | 2 | 2 | 1.5 | 0 | 1 |
| 5D5MEW+ | D | 5 | M | EW+ | 0 | 0 | 0.5 | 0 | 1.5 | 2 | 1 | 0 | 0 | 0 | 0 | 0.5 |
| 6D5MEW+ | D | 5 | M | EW+ | 0.5 | 0 | 0 | 0 | 0 | 0 | 0 | 0 | 0.5 | 0 | 0 | 0 |
| 1L0AEW+ | L | 0 | A | EW+ | 0 | 0.5 | 0 | 0 | 0 | 0 | 1 | 0 | 0 | 0 | 0 | 0 |
| 2L0AEW+ | L | 0 | A | EW+ | 0 | 0 | 0 | 1 | 0.5 | 0 | 0 | 0 | 0.5 | 0 | 0 | 0 |
| 3L0AEW+ | L | 0 | A | EW+ | 0 | 0 | 0 | 1 | 1.5 | 0 | 2 | 0 | 0 | 0 | 0 | 1 |
| 4L0AEW+ | L | 0 | A | EW+ | 0 | 0 | 0 | 0 | 0 | 0 | 0 | 0 | 0 | 0 | 0 | 0 |
| 5L0AEW+ | L | 0 | A | EW+ | 0.5 | 0 | 0 | 0 | 0 | 0.5 | 3 | 0 | 0 | 0 | 0 | 0.5 |
| 6L0AEW+ | L | 0 | A | EW+ | 0 | 0 | 0 | 0 | 0 | 1 | 3 | 0.5 | 0 | 0 | 0 | 0 |
| 1L5AEW+ | L | 5 | A | EW+ | 0 | 0 | 0.5 | 0 | 0 | 0 | 1 | 0.5 | 0 | 0 | 0 | 0 |
| 2L5AEW+ | L | 5 | A | EW+ | 0 | 1 | 0 | 0 | 1 | 0 | 1 | 0 | 0 | 0 | 0 | 0 |
| 3L5AEW+ | L | 5 | A | EW+ | 0 | 0.5 | 0 | 0.5 | 0 | 0.5 | 3 | 0 | 0 | 0 | 0 | 0 |
| 4L5AEW+ | L | 5 | A | EW+ | 0 | 0 | 0.5 | 0.5 | 0 | 2 | 0 | 1 | 0 | 0 | 0 | 0 |
| 5L5AEW+ | L | 5 | A | EW+ | 0 | 1.5 | 0 | 1 | 1 | 1 | 0 | 0 | 0 | 0 | 0.5 | 0 |
| 6L5AEW+ | L | 5 | A | EW+ | 0 | 0 | 1 | 0.5 | 0 | 1 | 0 | 0 | 0 | 1 | 0 | 0 |
| 1L0MEW+ | L | 0 | M | EW+ | 0 | 0.5 | 0 | 0 | 0 | 0 | 1 | 0 | 0 | 0 | 0 | 0 |
| 2L0MEW+ | L | 0 | M | EW+ | 0 | 0 | 0 | 1.5 | 0 | 0 | 1.5 | 0 | 0 | 0 | 0 | 0 |
| 3L0MEW+ | L | 0 | M | EW+ | 0 | 0 | 0 | 0 | 0.5 | 0 | 0 | 0 | 0 | 0 | 0 | 1 |
| 4L0MEW+ | L | 0 | M | EW+ | 0 | 0 | 0 | 0 | 0.5 | 0 | 2 | 0 | 0 | 0 | 0.5 | 0 |
| 5L0MEW+ | L | 0 | M | EW+ | 0 | 0 | 0 | 0 | 0 | 0.5 | 0 | 0 | 0.5 | 0 | 0 | 0 |
| 6L0MEW+ | L | 0 | M | EW+ | 0 | 0 | 0 | 0 | 2.5 | 2 | 4 | 0 | 0 | 0 | 0 | 0 |
| 1L5MEW+ | L | 5 | M | EW+ | 0 | 0 | 1.5 | 2 | 1.5 | 0.5 | 1 | 1 | 0 | 1 | 0 | 0 |
| 2L5MEW+ | L | 5 | M | EW+ | 0 | 0 | 1 | 0 | 0 | 0 | 0 | 1 | 0 | 0 | 1 | 0.5 |
| 3L5MEW+ | L | 5 | M | EW+ | 0 | 0.5 | 0 | 2 | 1 | 0 | 0.5 | 1 | 0 | 0 | 2 | 0 |
| 4L5MEW+ | L | 5 | M | EW+ | 1 | 0 | 1 | 2 | 1.5 | 0 | 0 | 2 | 0 | 0 | 1 | 0 |
| 5L5MEW+ | L | 5 | M | EW+ | 0 | 0.5 | 0 | 0 | 0 | 0 | 0 | 0 | 0 | 0 | 0.5 | 0 |
| 6L5MEW+ | L | 5 | M | EW+ | 0 | 0 | 0 | 0 | 2.5 | 1 | 1 | 0.5 | 0 | 0 | 0 | 0 |

**Supplementary Table S3** Raw data on effects of light pollution on earthworms and ragweed. Earthworm activity assessed by surface cast production (number of surface casts pot^-1^) on individual sampling days given in ordinal date. Abbreviations: LP…light pollution, SD…sowing depth, PS…plant species, EW…earthworms.

|  |  |  |  |  | **Sampling days** | | | | | | | | | | | |
| --- | --- | --- | --- | --- | --- | --- | --- | --- | --- | --- | --- | --- | --- | --- | --- | --- |
| **Pot code** | **LP** | **SD** | **PS** | **EW** | **103** | **105** | **108** | **110** | **113** | **116** | **122** | **126** | **133** | **134** | **137** | **141** |
| 1D0AEW+ | D | 0 | A | EW+ | 1 | 0 | 0 | 0 | 0 | 1 | 1 | 1 | 0 | 0 | 0 | 0 |
| 2D0AEW+ | D | 0 | A | EW+ | 1 | 0 | 0 | 1 | 0 | 1 | 1 | 2 | 0 | 0 | 1 | 0 |
| 3D0AEW+ | D | 0 | A | EW+ | 3 | 1 | 2 | 2 | 1 | 3 | 3 | 2 | 1 | 0 | 0 | 1 |
| 4D0AEW+ | D | 0 | A | EW+ | 0 | 0 | 1 | 1 | 1 | 2 | 1 | 1 | 1 | 0 | 1 | 0 |
| 5D0AEW+ | D | 0 | A | EW+ | 1 | 0 | 2 | 1 | 1 | 3 | 4 | 1 | 1 | 0 | 0 | 2 |
| 6D0AEW+ | D | 0 | A | EW+ | 2 | 0 | 1 | 1 | 2 | 1 | 2 | 2 | 0 | 0 | 0 | 1 |
| 1D5AEW+ | D | 5 | A | EW+ | 0 | 0 | 0 | 1 | 0 | 1 | 2 | 3 | 0 | 1 | 1 | 0 |
| 2D5AEW+ | D | 5 | A | EW+ | 0 | 0 | 1 | 2 | 0 | 3 | 3 | 3 | 0 | 0 | 0 | 0 |
| 3D5AEW+ | D | 5 | A | EW+ | 1 | 0 | 1 | 1 | 0 | 1 | 2 | 3 | 0 | 0 | 0 | 0 |
| 4D5AEW+ | D | 5 | A | EW+ | 2 | 0 | 2 | 1 | 0 | 2 | 4 | 2 | 0 | 0 | 0 | 1 |
| 5D5AEW+ | D | 5 | A | EW+ | 1 | 0 | 1 | 1 | 0 | 2 | 2 | 2 | 0 | 0 | 1 | 0 |
| 6D5AEW+ | D | 5 | A | EW+ | 0 | 0 | 2 | 2 | 0 | 1 | 2 | 0 | 0 | 0 | 0 | 0 |
| 1D0MEW+ | D | 0 | M | EW+ | 2 | 0 | 2 | 2 | 1 | 2 | 2 | 2 | 0 | 0 | 1 | 1 |
| 2D0MEW+ | D | 0 | M | EW+ | 2 | 0 | 1 | 0 | 0 | 2 | 2 | 2 | 0 | 0 | 1 | 0 |
| 3D0MEW+ | D | 0 | M | EW+ | 0 | 0 | 1 | 0 | 1 | 1 | 2 | 2 | 0 | 0 | 1 | 0 |
| 4D0MEW+ | D | 0 | M | EW+ | 2 | 0 | 2 | 3 | 1 | 3 | 3 | 4 | 1 | 0 | 1 | 2 |
| 5D0MEW+ | D | 0 | M | EW+ | 2 | 1 | 1 | 1 | 2 | 2 | 2 | 2 | 0 | 0 | 0 | 0 |
| 6D0MEW+ | D | 0 | M | EW+ | 0 | 0 | 1 | 1 | 0 | 1 | 2 | 0 | 0 | 0 | 0 | 0 |
| 1D5MEW+ | D | 5 | M | EW+ | 0 | 1 | 1 | 1 | 0 | 1 | 1 | 2 | 0 | 0 | 0 | 0 |
| 2D5MEW+ | D | 5 | M | EW+ | 1 | 0 | 1 | 1 | 1 | 1 | 2 | 2 | 0 | 0 | 0 | 0 |
| 3D5MEW+ | D | 5 | M | EW+ | 0 | 0 | 1 | 1 | 0 | 2 | 2 | 2 | 0 | 0 | 0 | 1 |
| 4D5MEW+ | D | 5 | M | EW+ | 0 | 0 | 0 | 0 | 0 | 1 | 2 | 0 | 0 | 0 | 0 | 0 |
| 5D5MEW+ | D | 5 | M | EW+ | 0 | 0 | 0 | 1 | 0 | 1 | 0 | 0 | 0 | 0 | 0 | 0 |
| 6D5MEW+ | D | 5 | M | EW+ | 0 | 0 | 0 | 0 | 0 | 0 | 0 | 0 | 0 | 0 | 0 | 0 |
| 1L0AEW+ | L | 0 | A | EW+ | 1 | 0 | 0 | 0 | 0 | 0 | 1 | 0 | 0 | 0 | 0 | 0 |
| 2L0AEW+ | L | 0 | A | EW+ | 2 | 0 | 1 | 0 | 0 | 0 | 0 | 1 | 0 | 0 | 0 | 0 |
| 3L0AEW+ | L | 0 | A | EW+ | 1 | 0 | 1 | 0 | 0 | 2 | 1 | 2 | 0 | 0 | 0 | 0 |
| 4L0AEW+ | L | 0 | A | EW+ | 0 | 0 | 0 | 1 | 0 | 0 | 2 | 0 | 0 | 0 | 0 | 0 |
| 5L0AEW+ | L | 0 | A | EW+ | 0 | 0 | 0 | 1 | 0 | 0 | 0 | 1 | 0 | 0 | 0 | 0 |
| 6L0AEW+ | L | 0 | A | EW+ | 1 | 0 | 0 | 1 | 0 | 0 | 1 | 2 | 0 | 0 | 0 | 0 |
| 1L5AEW+ | L | 5 | A | EW+ | 1 | 0 | 0 | 1 | 0 | 1 | 0 | 1 | 0 | 0 | 0 | 0 |
| 2L5AEW+ | L | 5 | A | EW+ | 0 | 0 | 2 | 2 | 0 | 1 | 1 | 2 | 0 | 0 | 0 | 0 |
| 3L5AEW+ | L | 5 | A | EW+ | 0 | 0 | 1 | 1 | 0 | 0 | 3 | 1 | 0 | 0 | 0 | 0 |
| 4L5AEW+ | L | 5 | A | EW+ | 1 | 0 | 0 | 0 | 0 | 1 | 2 | 1 | 0 | 0 | 0 | 0 |
| 5L5AEW+ | L | 5 | A | EW+ | 0 | 0 | 1 | 0 | 0 | 0 | 2 | 0 | 0 | 0 | 0 | 0 |
| 6L5AEW+ | L | 5 | A | EW+ | 1 | 0 | 2 | 1 | 0 | 1 | 1 | 2 | 0 | 0 | 0 | 0 |
| 1L0MEW+ | L | 0 | M | EW+ | 4 | 0 | 1 | 1 | 0 | 1 | 2 | 3 | 0 | 0 | 0 | 0 |
| 2L0MEW+ | L | 0 | M | EW+ | 1 | 0 | 0 | 3 | 0 | 0 | 2 | 1 | 0 | 0 | 0 | 0 |
| 3L0MEW+ | L | 0 | M | EW+ | 1 | 0 | 0 | 1 | 0 | 0 | 0 | 0 | 0 | 0 | 0 | 0 |
| 4L0MEW+ | L | 0 | M | EW+ | 2 | 0 | 2 | 2 | 0 | 0 | 2 | 0 | 0 | 0 | 0 | 0 |
| 5L0MEW+ | L | 0 | M | EW+ | 1 | 0 | 2 | 2 | 0 | 1 | 1 | 2 | 0 | 0 | 0 | 0 |
| 6L0MEW+ | L | 0 | M | EW+ | 0 | 0 | 1 | 1 | 0 | 1 | 2 | 3 | 0 | 0 | 0 | 0 |
| 1L5MEW+ | L | 5 | M | EW+ | 1 | 1 | 0 | 0 | 1 | 1 | 2 | 1 | 0 | 0 | 0 | 0 |
| 2L5MEW+ | L | 5 | M | EW+ | 1 | 0 | 0 | 0 | 0 | 1 | 1 | 1 | 0 | 0 | 0 | 0 |
| 3L5MEW+ | L | 5 | M | EW+ | 1 | 0 | 1 | 2 | 0 | 0 | 1 | 1 | 0 | 0 | 0 | 0 |
| 4L5MEW+ | L | 5 | M | EW+ | 2 | 0 | 2 | 2 | 0 | 0 | 2 | 2 | 0 | 0 | 0 | 0 |
| 5L5MEW+ | L | 5 | M | EW+ | 0 | 0 | 0 | 0 | 0 | 0 | 0 | 0 | 0 | 0 | 0 | 0 |
| 6L5MEW+ | L | 5 | M | EW+ | 1 | 0 | 1 | 0 | 0 | 1 | 2 | 2 | 0 | 0 | 0 | 0 |
